# Association of First-Week Nutrient Intake and Extrauterine Growth Restriction in Moderately Preterm Infants: A Regional Population-Based Study

**DOI:** 10.3390/nu13010227

**Published:** 2021-01-14

**Authors:** Marine Baillat, Vanessa Pauly, Gina Dagau, Julie Berbis, Farid Boubred, Laurence Fayol

**Affiliations:** 1 Department of Neonatology, Hôpital La Conception, Hôpitaux Universitaires de Marseille, 13005 Marseille, France; marine.baillat@gmail.com (M.B.); farid.boubred@ap-hm.fr (F.B.); 2 Research Unit EA 3279 and Department of Public Health, Aix-Marseille University, 13005 Marseille, France; vanessa.pauly@ap-hm.fr (V.P.); julie.berbis@ap-hm.fr (J.B.); 3 Service de Néonatologie, Centre Hospitalier de Martigues, 13698 Martigues CEDEX, France; gdagau@gmail.com; 4 Aix-Marseille Université, C2VN, INRAE, INSERM, 13005 Marseille, France; 5 Réseau Périnatal Méditerranée (PACA-Corse-Monaco), 13015 Marseille, France

**Keywords:** premature infant, nutrition, growth, moderately preterm, extrauterine growth restriction, energy, protein

## Abstract

The purpose of this study was to determine the influence of first-week nutrition intake on neonatal growth in moderate preterm (MP) infants. Data on neonatal morbidity and nutrition intake on day of life 7 (DoL7) were prospectively collected from 735 MP infants (32^0/7^–34^6/7^ weeks gestational age (GA)). Multivariable regression was used to assess the factors associated with extrauterine growth restriction (EUGR) defined as a decrease of more than 1 standard deviation (*SD*) in the weight *z*-score during hospitalization. Mean (*SD*) gestational age and birth weight were 33.2 (0.8) weeks and 2005 (369) g. The mean change in the weight *z*-score during hospitalization was −0.64 *SD*. A total of 138 infants (18.8%) had EUGR. Compared to adequate growth infants, EUGR infants received 15% and 35% lower total energy and protein intake respectively (*p* < 0.001) at DoL7. At DoL7, each increase of 10 kcal/kg/d and 1 g/kg/d of protein was associated with reduced odds of EUGR with an odds ratio of 0.73 (95% CI, 0.66–0.82; *p* < 0.001) and 0.54 (0.44–0.67; *p* < 0.001), respectively. Insufficient energy and protein intakes on DoL7 negatively affected neonatal growth of MP infants. Nutritional support should be optimized from birth onwards to improve neonatal weight growth.

## 1. Introduction

Preterm birth, defined as birth before 37 full weeks, remains the leading cause of death and complications in the neonatal period. Infants born moderately preterm, with gestational age (GA) between 32 and 34 completed weeks, account for 20% to 30% of preterm births [1,2]. Although serious morbidities are rare in moderate preterm (MP) infants, the rate of respiratory disease is about 25% to 30%, the rate of neonatal infection is about 10% to 15%, the rate of hypoglycemia is about 15%, and the rate of jaundice is about 60% [3,4,5,6,7]. MP infants have elevated risk of hospital readmission and have a 2-fold increased risk of neurodevelopment disabilities at 2 years than term infants [5,8,9]. They have unique and often unrecognized medical vulnerabilities and are frequently incorrectly considered to be healthy infants.

Early nutrition is an important determinant of neonatal growth, neonatal morbidity/mortality, and psychomotor development in preterm infants [10]. Growth and nutrition determinants have been largely studied in very preterm (VPT) and extremely preterm (EPT) infants; postnatal growth failure is known to affect short- and long-term health and neurocognitive functions [11,12,13,14,15,16]. However, little is known about their influence on the health outcomes of MP infants. Recommended intakes, based on fetal needs, are proportionally higher in immature infants and should ideally be met within the first postnatal week [17,18]. Compared to term-born infants, MP infants are born at a time of rapid development when nutrient requirements are high; however, their nutritional intake is poorly managed [19]. In contrast to VPT/EPT infants, MP infants are less frequently parenterally fed [20] and predominantly receive their nutrition intake enterally. There is a lack of information and consistency in the nutritional management of this vulnerable and neglected population. Studies have shown that MP infants are at a risk of postnatal growth failure, with significant variations in feeding practices among neonatal intensive care units (NICUs) [21,22,23]. Recent reports have shown that the mean daily protein intake in the first week enhances weight and head circumference growth in MP infants, as for VPT and EPT infants [24]. However, data are scarce, and further studies are needed to better investigate the role of macronutrients, including energy and protein, on neonatal growth.

The purposes of this multicenter observational study were to (1) describe neonatal growth and variation in nutrition practices and (2) determine the influence of first-week energy and protein intake on neonatal growth in a cohort of MP infants.

## 2. Patients and Methods

### 2.1. Study Design and Population

This study was a population-based observational, non-interventional, cohort study. This study was part of a health care quality improvement project that aims to improve neonatal health care of preterm infants in a regional perinatal network. Because this was a non-interventional study, signed parental consent was not required. Parents were informed of the study and could oppose the collection of data on their infants. The infants’ records were anonymized for analysis. This study was declared to the Commission Nationale de l’Informatique et des Libertés (CNIL, number 1979318) and was approved by the scientific committee of the regional perinatal health network.

We included all MP infants born between January 1, 2017, and December 31, 2017, with GA from 32^0/7^ weeks to 34^6/7^ weeks, and who were admitted to the neonatal units of the region PACA-Corse-Monaco in the southeast of France. The region PACA-Corse-Monaco records nearly 61,000 births per year and includes three level 3 NICUs and eighteen level 2 neonatal units. All twenty-one neonatal units participated in this study. Infants who died before discharge from neonatal units, those with major congenital and genetic abnormalities, and those with a lack of data regarding growth parameters (at birth, day 7, and discharge) were excluded.

### 2.2. Nutrition and Growth Parameters

Nutrition intake were collected and calculated at day of life 1 (DoL1) and 7 (DoL7) and at discharge (D/C). Volume, energy, protein, lipid, and glucose intakes were calculated from daily enteral feeding (including human milk (HM) and infant formulas) and parenteral nutrition. HM fortification quantities and the day of initiation were also noted. Donor mature HM was assumed to comprise 1.2 g of protein, 3.2 g of fat and 7.8 g of carbohydrate per 100 mL, while moderately preterm HM was considered to contain 1.9 g of protein, 4.8 g of fat and 7.5 g of carbohydrate per 100 mL [25]. Parenteral nutrition length and support and use of a central venous line (including umbilical venous catheter) were also noted. We collected data on body weight (BW), height (H), and head circumference (HC) at DoL1, DoL7, and D/C. Small for gestational age (SGA), defined as a birth weight less than −1.28 *SD* below the mean weight for GA, and the *z*-scores for BW, H, and HC at DoL1, DoL7, and D/C, and their variations over time (delta z-score), were calculated using Fenton growth curves (https://peditools.org/fenton2013). Extrauterine growth restriction (EUGR) was defined as a drop of delta BW *z*-score of more than 1 *SD* from birth (DoL1) to D/C from neonatal units [26]. The target values for energy and protein intakes in MP infants at the end of first week were at least 90 kcal/kg/day and at least 2.5 g/kg/day, respectively [19,27,28]. As this was a non-interventional study, nutritional management was left to the discretion of the medical staff.

### 2.3. Data Collection

Obstetric and neonatal data were collected from the infants’ medical records. Data on maternal age, multiple births, antenatal steroid therapy, obstetrical complications, and mode of delivery were also collected. GA was calculated according to the date of the last menstrual period and/or early obstetrical ultrasound. Neonatal morbidity included severe respiratory distress syndrome (RDS), defined as the need for endotracheal surfactant therapy; nasal continuous positive airway pressure (nCPAP) use; necrotizing enterocolitis (NEC) ≥ stage II according to Bell’s definition; early-onset infection (in the first three days); severe cerebral injury (intraventricular hemorrhage ≥ stage III according to Papile’s definition or periventricular leukomalacia diagnosed on head ultrasound or cerebral magnetic resonance imagery); bronchopulmonary dysplasia, defined as the need for oxygen or respiratory support at day 28; catheter-related bloodstream infections (CRBIs), defined as at least one positive blood culture associated with clinical manifestations or antibiotic use for at least 5 days; and length of hospital stay. The neonatal units were divided into three groups according to the number of MP infants admitted during the study period as follows: less than 20; 20 to 50; and more than 50 infants.

### 2.4. Statistical Analysis

We first described the population and then compared groups with and without EUGR. We performed statistical analyses for such comparisons using the chi-squared or Fisher’s exact tests for qualitative variables and Student’s *t*-test or Kruskal–Wallis non-parametric tests (when conditions for Student’s *t*-test were not satisfied) for quantitative variables. Variables with *p*-values < 0.20 in bivariable analyses were included in a step-by-step backward multivariable analysis in order to identify the factors associated with EUGR. We performed a multivariable generalized logistic regression (PROC GLIMMIX in SAS ®, SAS Institute, Cary, NC, USA), with the place of hospitalization as a random effect to take into account correlation of data due to the center effect. Confounding factors were eliminated and total energy intake at DoL7 was retained to represent nutrient intake in a first model, and total protein intake at DoL7 in a second model. The results are presented as odds ratios (ORs) and their 95% confidence intervals (CIs). In this study, *p*-values < 0.05 were considered statistically significant. The analyses were performed using IBM SPSS Statistics for Windows, IBM Company, Chicago, IL, USA, Version 20.0, and SAS^®^, Version 9.4.

## 3. Results

During the study period, a total of 835 MP infants with GA 32^0/7^ and 34^6/7^ weeks born between 1 January 2017, and 31 December 2017, were admitted to the 21 participating neonatal units. Six infants died before discharge, two had major congenital abnormalities, and 92 had missing data regarding the primary outcome (EUGR). Finally, 735 MP infants were included. The mean (*SD*) GA and birth weight were 33.2 (0.8) weeks and 2005 (369) g, respectively, with nearly half of the infants born with GA at 34 weeks (Table 1). In this study, 85 infants (11.6%) were born SGA, 57 (7.7%) had RDS treated with surfactant, and 11 (1.5%) had NEC stage ≥ 2. The rate of CRBI was low (less than 1%), and 72% of the population received antenatal steroids.

### 3.1. Growth and Nutrition Parameters

In the overall population, the mean BW *z*-score decreased from −0.31 (0.8) at birth (DoL1) to −0.95 (0.8) at discharge (D/C) (*p* < 0.001). This drop mostly occurred within the first week of life, with the weight *z*-score decreasing from −0.31 (0.8) at DoL1 to −0.92 (0.7) at DoL7 (*p* < 0.001) (Table 2). No catch-up growth was observed thereafter. During the study period, 138 (18.8%) infants had EUGR. Compared to infants with adequate growth rates, EUGR infants required a mean of 3.5 additional days to regain DoL1 BW (*p* < 0.01) and displayed a slower growth rate after DoL7 (Table 2).

In the overall population, the mean enteral intake was 132 (32) mL/kg at DoL7. HM remained the main source of enteral feeding (54.4%, *n* = 400) but fortification was prescribed in only 57.5% of the infants fed HM. HM was less frequently fortified in EUGR infants compared to infants with appropriate growth (41.3% and 62.3%, respectively, *p* < 0.001). The milk was obtained from donated milk at day 7 in 17.8% of cases (*n* = 71). In the EUGR breast-fed infants, 22.8% of HM came from donated milk versus 6.2% in the no EUGR breast-fed infants (*p* = 0.14). At D/C, 55% of the infants were breastfed, and 37% of them received infant formula as a complement.

At DoL1, 436 (59.4%) infants received parenteral nutrition (PN), with solutions free of proteins and lipids in 12.5% and 68% of infants, respectively. At DoL7, 94 (12.8%) infants were receiving PN, but 31.1% remained free of lipids. In this study population, 61.3% and 68.4% of 1-week-old infants received more than 90 kcal/kg/day of energy and more than 2.5 g/kg/day of proteins, respectively (Supplementary Material, Table S1). Table 3 shows growth and nutrition parameters according to GA.

Bodyweight *z*-score at different points and variation between DoL1 and D/C were not different between GA groups. Preterm infants born with GA 32 weeks were more likely to have EUGR than 34-week-old infants (*p* < 0.01). Total DoL7 energy and protein intake were significantly lower in most immature infants as well.

### 3.2. Early Nutrient Intake and EUGR Rate

Compared to infants with adequate growth rates, infants with EUGR were more likely to be born with a lower GA (*p* < 0.001), have a higher birth weight *z*-score (*p* < 0.001), and have RDS (*p* < 0.001) (Table 1). At DoL7, they received more frequent PN (18.8% vs. 11.4%, *p* < 0.001) but with 22% lower energy amounts (*p* < 0.05). At the end of the first week of life, EUGR infants received lower enteral volume (120 vs. 135 mL/kg/d, *p* < 0.001), with 16% less enteral energy (*p* < 0.001), and 24% less enteral protein (*p* < 0.001) intake. Total (enteral and parenteral) energy (−15%, *p* < 0.001) and protein (−35%, *p* < 0.001) intake at DoL7 was significantly lower in EUGR infants (Table 2). After adjusting for principal perinatal factors (GA, BW *z*-score, RDS, nCPAP use, CRBI, severe brain injuries, length of hospital stay, neonatal unit volume, and PN duration), early nutrient intake was associated with EUGR (Table 4). Model 1 shows that for each 10 kcal/kg/d increase energy intake at DoL7 there was a lower odds of EUGR (OR (95% CI) = 0.73 (0.66–0.82); *p* < 0.001). In model 2, each 1 g/kg/d increase in protein intake at DoL7 was associated with reduced odds of EUGR of 46% with OR (95% CI) = 0.54 (0.44–0.67) (*p* < 0.001). While elevated weight *z*-score at birth and respiratory disease were risk factors, admission to a neonatal unit with one of the highest admission volumes was a preventive factor of EUGR (OR (95% CI) = 0.40 (0.18–0.91), admission number > 50, Model 1).

## 4. Discussion

In this population-based study of MP infants, we found that approximately 19% of infants had EUGR that mostly occurred during the first week of life. Nutritional practices varied between neonatal units, which led to wide ranges in early protein and energy intake. In this study, about 60% of infants did not reach the recommended targeted nutrition intake at the end of the first week of life. We also found that early energy and protein intake positively influenced neonatal growth.

Growing evidence suggests that MP infants experience postnatal growth failure during the neonatal period [19]. In contrast to VPT and EPT infants, few studies have described neonatal growth in MP infants. In a single study of 235 infants with a mean GA of 33 weeks, Gerritsen et al. reported a decrease of 0.4 in the weight *z*-score from birth to D/C [24]. In a regional population study of 450 MP infants in the US, Blackwell et al. reported a weight *z*-score decrease of 0.67 [21], a difference similar to that observed in our population study with a larger size. Neither study reported EUGR rates. We also found that growth failure mostly occurred during the first week of life without significant catch-up before D/C. The growth rate continued to be slower in EUGR infants and slightly accelerated in appropriate-growth infants. Aside from nutrition intake, we found that being bigger at birth and RDS were each associated with EUGR. The possibility that nutritional support was inadequate to the metabolic demands of bigger infants cannot be excluded. Gestational age was a limited factor in our study, the growth pattern of infants was the same regardless of GA. We can hypothesize that nutrition support was insufficient in 34-week-old infants. Taken together, these findings suggest that postnatal growth failure is of concern in MP infants, since it may expose them to neonatal morbidity and may later affect long-term neurocognitive outcomes [26,29]. Pediatricians caring for these infants should be aware of this risk and implement appropriate nutritional strategies.

The influence of early nutrition on neonatal growth has been little investigated in MP infants. Most studies have shown that early deficits in protein and energy intake during the first 2 weeks of life affect neonatal growth and long-term neurocognitive functions in VPT and EPT infants [17,30,31,32]. In a population of MP infants, Gerritsen et al. showed that every 1 g/kg/d increase in mean daily protein intake in the first week resulted in a weight *z*-score increase of 0.34 (95% CI, 0.14–0.53) [24] at term corrected age; only half of the infants reached the recommended protein intake at DoL7. We also found a 46% reduction in EUGR for each 1 g/kg/d increase protein intake at DoL7. Energy plays a determinant role as well. We observed for each 10 kcal/kg/d increase in energy at the end of the first week of life a 27% lower odds of EUGR at D/C (OR 0.73; 95% CI, 0.66–0.82). In a similar population, Yagasaki et al. showed that the cumulative energy intakes during the first weeks of life were inversely correlated with the body loss rate [33]. These findings highlight the determinant role of early nutrition strategies on neonatal growth in MP infants, who mostly failed to reach the recommended target nutrition intake at the end of the first week of life [22,24,34]. In our study, only 60% to 70% of infants received the targeted energy (90–120 kcal/kg/d) and protein intake (2.5–3.5 /kg/d) at the end of the first week [27,28]. The percentage of MP infants reaching these targets also varied greatly between neonatal units, and these differences indicate a potential for improving nutritional support. These findings show that the initiation of adequate nutrition was insufficient in most MP infants. Pediatricians should perform daily monitoring of body weight and carefully manage nutrition from birth onwards by promoting early and aggressive nutrition strategies; targeted nutrition intake should be achieved in the first week of life. Such strategies, through limiting excessive weight loss after birth and promoting early catch-up growth, may prevent EUGR.

Our findings suggest that optimizing enteral feeding from birth onwards may improve neonatal growth in MP infants. As observed in our study, enteral feeding represented the principal nutritional support and accounted for a significant part of macronutrient intake at the end of the first week. Indeed, in this preterm population, PN needs are limited and the risks of parenteral nutrition-related complications (venous thrombosis, infections, pain) outweigh those of enteral nutrition. Few underlying pathways can be discussed. Our study revealed insufficient fortification of HM at DoL7. HM was the principal enteral feeding, with about 55% of the infants being breastfed during hospitalization and 55% remained so at discharge; however, only 60% of breastfed infants received a fortifier in HM at the end of first week. The volume of enteral feeding was also lower in EUGR infants, which would have provided less energy and protein intakes. Fortification is commonly discontinued when the infant begins to suckle the breast, leading to a reduced protein intake during this transition period, while MP infants’ needs are higher than those of term newborn to meet their metabolic demands [35]. All of these nutritional practices may overexpose infants to early nutritional deficits and may explain why the growth rates continued to be slow in EUGR infants during their hospital stay. To prevent EUGR, nutritional practices should include (1) optimal support of breastfeeding mothers because of the beneficial effects of human milk both in the short-term (lower risk of infection, NEC, feed tolerance) and long-term outcomes (neurodevelopmental outcomes, cardiovascular risks, and bone health); (2) supply HM from DoL1 with a rapid increase in enteral volumes if well tolerated; (3) early use of fortification; (4) use of preterm formula, if required, as a supplement during the transition to suckling; and (5) use PN, if required, with protein and lipids administration from birth onwards according to recent international guidelines [36,37,38,39,40].

This study had some limitations. Data on daily nutrition intake and growth parameters for different times of hospital stay were not available; thus, we could not evaluate the first-week cumulative macronutrient intake and growth velocity. We were also un-able to investigate factors influencing length and head circumference growth due to large amounts of missing data. Another limitation was the accurate estimation of metabolizable nutritional intake in enteral nutrition. There was high variation between the donors’ HM and own breastfeeding, inter-individual HM composition and in the expressed HM composition because of the collection and storage conditions as well as the stage of lactation. Further studies may include macronutrient dosage in HM to better evaluate the role of each macronutrient. The infants were admitted to neonatal units of different sizes and levels of care. We found that infants admitted to the units receiving the largest numbers of MP infants each year were less likely to be EUGR. Neonatal volume activities were considered to be an indicator of the quality of care, with low morbidity and mortality observed in high-volume neonatal units [41]. The variable we used as a surrogate marker of medical staff experience needs to be better defined and adjusted to the center level of care and perinatal/neonatal morbidities. The strength of this study was that it revealed the growth and nutrition support differences in the largest known cohort of MP infants. This population-based study revealed what happens in “real world” conditions, and we believe our findings could be generalizable.

In conclusion, MP infants are prone to developing extrauterine growth restriction associated with suboptimal early nutrient intake. Medical staff should be aware of the risk of postnatal growth failure, which may occur within the first days after birth, and should optimize enteral feeding from birth onwards to prevent energy and protein deficits. Extrauterine growth restriction may be prevented by improving protein and energy intake during the first week of life.

## Figures and Tables

**Table 1:** Obstetrical and neonatal characteristics of study population by EUGR.

	Overall Population *n* = 735	EUGR *n* = 138	No EUGR *n* = 597	*p*
**Obstetrical characteristics**				
**Cesarean delivery, *n* (%)**	344 (46.5)	67 (48.5)	275 (46.1)	0.61
**Prenatal steroids, *n* (%)**	531 (72.2)	99 (71.7)	432 (72.3)	0.81
**Spontaneous preterm labor, *n* (%)**	429 (58.3)	91 (65.9)	338 (56.6)	0.04
**Pre-eclampsia, *n* (%)**	178 (24.2)	25 (18.1)	153 (25.6)	0.06
**Premature rupture of membrane, *n* (%)**	93 (12.6)	13 (9.4)	80 (13.4)	0.2
**Multiple pregnancy, *n* (%)**	228 (31)	43 (31.1)	185 (30.9)	0.96
**Neonatal characteristics**				
**GA (weeks), mean ± *SD***	33.2 ± 0.8	33.0 ± 0.8	33.3 ± 0.8	<0.01
**GA**				0.003
**32 weeks, *n* (%)**	170 (23.1)	47 (34)	123 (20.6)	
**33 weeks, *n* (%)**	200 (27.2)	35 (25.3)	165 (27.6)	
**34 weeks, *n* (%)**	365 (49.7)	56 (40.6)	309 (51.7)	
**Sex male, *n* (%)**	419 (56.8)	85 (61.6)	334 (55.8)	0.22
**SGA, *n* (%)**	85 (11.6)	11 (8.0)	74 (12.4)	0.14
**RDS, *n* (%)**	57 (7.7)	19 (13.7)	38 (6.3)	<0.01
**nCPAP, *n* (%)**	351 (47.7)	84 (60.8)	267 (44.7)	<0.01
**Early onset infection, *n* (%)**	23 (3.1)	5 (3.6)	18 (3.0)	0.78
**NEC stage ≥ II, *n* (%)**	11 (1.5)	3 (2.1)	8 (1.3)	0.44
**Severe brain injuries, *n* (%)**	4 (0.5)	0 (0)	4 (0.7)	0.99
**Central line, *n* (%)**	131 (17.8)	28 (20.3)	103 (17.2)	0.4
**CRBI, *n* (%)**	6 (0.8)	3 (2.2)	3 (0.5)	0.08
**Length of stay (days), mean ± *SD***	22.6 ± 11.0	25.0 ± 11.1	22.1 ± 11.0	<0.01
**NICU Level**				
**Level 2, *n* (%)** **Level 3, *n* (%)**	528 (71.8) 190 (25.8)	104 (75.3) 32 (23.1)	424 (71.0) 158 (26.4)	0.3 0.42
**NICU admission volume** **<20 MP infants, *n* (%)** **20–50 MP infants, *n* (%)** **>50 MP infants, *n* (%)**	80 (100) 122 (100) 533 (100)	21 (26.2) 26 (21.3) 91 (17.1)	59 (73.7) 96 (78.7) 442 (82.9)	0.1

EUGR: extrauterine growth restriction; GA: gestational age; *SD*: standard deviation; SGA: small for gestational age; RDS: respiratory distress syndrome; nCPAP: nasal continuous positive airway pressure; NEC: necrotizing enterocolitis; CRBI: catheter-related bloodstream infection; NICU Level: neonatal intensive care unit (level 2 and 3); NICU admission volume: MP infants admitted during the study period.

**Table 2:** Nutrients and growth of the study population according to EUGR.

	Overall Population *n* = 735	EUGR *n* = 138	No EUGR *n* = 597	*p*
**Growth characteristics**				
**Weight (g), mean ± *SD***				
**DoL1**	2004 ± 370	2099 ± 401	1982 ± 359	<0.01
**DoL7**	1952 ± 355	1979 ± 397	1945 ± 344	0.36
**D/C**	2437 ± 325	2390 ± 290	2448 ± 332	0.04
**Weight *z*-score, mean ± *SD***				
**DoL1**	−0.31 ± 0.8	0.03 ± 0.8	−0.39 ± 0.7	<0.001
**DoL7**	−0.92 ± 0.7	−0.76 ± 0.8	−0.96 ± 0.7	<0.01
**D/C**	−0.95 ± 0.8	−1.20 ± 0.9	−0.9 ± 0.7	<0.001
**Delta weight *z*-score DoL1-D/C, mean ± *SD***	−0.64 ± 0.4	−1.26 ± 0.3	−0.5 ± 0.3	<0.001
**Regaining BW (days), mean ± *SD***	9.1 ± 3.6	12 ± 4.6	8.5 ± 3	<0.001
**Nutrition data**				
**Breastfeeding at discharge, *n* (%)**	395 (53.7)	90 (65.0)	305 (51.0)	0.002
HM fortification, *n*/*N* * (%)	230/400 (57.5)	38/92 (41.3)	192/308 (62.3)	<0.001
**PN initiation, *n* (%)**	440 (59.8)	99 (71.7)	341 (57.1)	0.001
**PN duration (days), mean ± *SD***	6.1 ± 6.3	6.1 ± 4.5	6.1 ± 6.8	0.8
**Enteral feeding DoL7**				
**Volume intake (mL/kg), mean ± *SD***	132 ± 32	120 ± 32	135 ± 31	<0.001
**Energy intake (kcal/kg), mean ± *SD***	91 ± 25	79 ± 24	94 ± 25	<0.001
**Protein intake (g/kg), mean ± *SD***	2.8 ± 1.2	2.2 ± 1.1	2.9 ± 1.2	<0.001
**Lipid intake (g/kg), mean ± *SD***	4.4 ± 1.2	3.9 ±1.1	4.5 ± 1.2	<0.001
**Parenteral feeding DoL 7**				
**Number, *n* (%)**	94 (12.8)	26 (18.8)	68 (11.4)	0.01
**Energy intake (kcal/kg), mean ± *SD***	47 ± 23	39 ± 23	50 ± 23	0.04
**Protein intake (g/kg), mean ± *SD***	1.6 ± 0.9	2.2 ± 1.1	2.0 ± 0.9	0.10
**Lipid intake (g/kg), mean ± *SD***	1.3 ± 1.0	1.3 ± 0.9	1.4 ± 1.1	0.66
**Total nutrient DoL7**				
**Volume intake (mL/kg), mean ± *SD***	142 ± 25	131 ± 25	144 ± 25	<0.001
**Energy intake (kcal/kg), mean ± *SD***	96 ± 21	84 ± 21	99 ± 21	<0.001
**Protein intake (g/kg), mean ± *SD***	3.0 ± 1.1	2.0 ± 1.0	3.1 ± 1.1	<0.001
**Lipid intake (g/kg), mean ± *SD***	4.5 ± 1.1	4.0 ± 1.1	4.6 ± 1.1	<0.001

EUGR: extrauterine growth restriction for weight; *SD:* standard deviation; DoL: day of life; D/C: discharge; BW: birth weight; HM: human milk; * n: number of infants receiving HM fortification; N: total number of infants receiving HM; PN: parenteral nutrition.

**Table 3:** Nutrients and growth during the first week according to gestational age.

	32 Weeks *n* = 170	33 Weeks *n* = 200	34 Weeks *n* = 365	*p*
**Growth characteristics**				
**BW *z*-score**				
**DoL1, mean ± *SD***	−0.23 ± 0.80	−0.36 ± 0.80	−0.32 ± 0.77	0.33
**DoL7, mean ± *SD***	−0.89 ± 0.73	−0.94 ± 0.73	−0.92 ± 0.73	0.87
**D/C, mean ± *SD***	−0.92 ± 0.86	−0.98 ± 0.81	−0.96 ± 0.72	0.74
**Delta weight *z*-score DoL1-D/C, mean ± *SD***	−0.69 ± 0.55	−0.63 ± 0.43	−0.64 ± 0.38	0.41
**EUGR, *n* (%)**	47 (27.6)	35 (17.5)	56 (15.3)	<0.01
**Nutrition data**				
**PN initiation, *n* (%)**	142 (83.5)	146 (73.0)	146 (40.0)	<0.001
**PN duration (days), mean ± *SD***	7.3 ± 8.5	4.3 ± 4.8	1.9 ± 3.6	<0.001
**Total nutrient DoL7**				
**Volume intake (mL/kg), mean ± *SD***	142 ± 29	141 ± 28	139 ± 29	0.74
**Energy intake (kcal/kg), mean ± *SD***	91 ± 23	96 ± 19	98 ± 21	<0.01
**Protein intake (g/kg), mean ± *SD***	2.7 ± 1.2	3.0 ± 1.0	3.2 ± 1.1	<0.0001
**Lipid intake (g/kg), mean ± *SD***	4.1 ± 1.2	4.5 ± 1.1	4.7 ± 1.1	<0.0001
**Infants reaching nutritional targets at DoL7**				
**Total energy intakes > 90 kcal/kg/day, *n* (%)**	86 (50.6)	115 (57.5)	246 (68.2)	<0.01
**Total protein intake > 2.5 g/kg/day, *n* (%)**	94 (55.3)	136 (68)	273 (74.8)	<0.0001

BW: body weight; DoL: day of life; D/C: discharge; *SD*: standard deviation; EUGR: extrauterine growth restriction; PN: parenteral nutrition.

**Table 4:** Multivariable generalized logistic regression modeling EUGR with energy (Model 1) or protein (Model 2) intake at the end of first week.

	Model 1		Model 2	
	OR (CI 95%)	*p*	OR (CI 95%)	*p*
**DoL1 BW *z*-score (each 1 *SD* increase)**	1.59 (1.02–2.94)	<0.0001	2.21 (1.62–3.00)	<0.0001
**RDS**	2.15 (1.06–4.32)	0.03	*-*	-
**No nCPAP** **nCPAP < 24 h** **nCPAP ≥ 24 h**	-	-	Reference 1.26 (0.73–2.16) 2.02 (1.14–3.58)	0.40 0.01
**Length of hospital stay**	1.04 (1.02–1.06)	<0.0001	1.03 (1.01–1.06)	<0.0001
**NICU admission volume** **<20 MP infants, *n* (%)** **20–50 MP infants, *n* (%)** **>50 MP infants, *n* (%)**	Reference 0.53 (0.22–1.48) 0.40 (0.18–0.91)	0.25 0.02	Reference 0.35 (0.15–0.79) 0.55 (0.21–1.46)	0.01 0.22
**Total energy intake at DoL7** **(each 10 kcal/kg/day increase)**	0.73 (0.66–0.82)	<0.001	*-*	-
**Total protein intake at DoL7** **(each 1g/kg/day increase)**	-	-	0.54 (0.44–0.67)	<0.0001

EUGR: extrauterine growth restriction for weight; Model 1: multivariate generalized logistic regression model assessing EUGR with energy; Model 2: multivariate generalized logistic regression model assessing EUGR with protein; OR: odd ratio; CI: confidence interval; DoL, day of life; BW: body weight; RDS: respiratory distress syndrome; nCPAP: nasal continuous positive airway pressure; NICU admission volume: number of MP infants admitted in neonatal intensive care unit during the study period.

## Supplementary Materials

The following are available online at https://www.mdpi.com/2072-6643/13/1/227/s1: Table S1: Target of protein and energy intakes reached at the end of first week according to neonatal centers.
