# Supplementary material for: Association of First-Week Nutrient Intake and Extrauterine Growth Restriction in Moderately Preterm Infants: A Regional Population-Based Study
Source: Nutrients. 2021 Jan 14;13(1):227. doi: 10.3390/nu13010227 (PMC7830065; doi:10.3390/nu13010227)
Supplement: Supplementary file 1 [file nutrients-13-00227-s001.pdf]

Supplementary Material

Table S1: Target of protein and energy intakes reached at the end of first week according to neonatal centers.

	Proteins at DoL 7 > 2.5g/kg/day, % (n)	Energy at DoL 7 > 90 kcal/kg/day, %(n)
**Neonatal centers (n)**		
**A (2)**	50 (1)	0 (0)
**B (3)**	66.6 (2)	66.6 (2)
**C (5)**	80 (4)	100 (5)
**D (5)**	80 (4)	60 (3)
**E (9)**	66.6 (6)	88.8 (8)
**F (9)**	44.4 (4)	33.3 (3)
**G (9)**	77.7 (7)	77.7 (7)
**H (11)**	54.5 (6)	45.4 (5)
**I (12)**	75 (9)	58.3 (7)
**J (15)**	86.6 (13)	80 (12)
**K (24)**	54.2 (13)	37.5 (9)
**L (27)**	70.3 (19)	66.6 (18)
**M (34)**	47.1 (16)	35.3 (12)
**N (37)**	70.3 (26)	64.8 (24)
**O (49)**	87.7 (43)	79.5 (39)
**P (55)**	81.8 (45)	74.5 (41)
**Q (66)**	81.8 (54)	77.2 (51)
**R (74)**	70.2 (52)	64.8 (48)
**S (85)**	80 (68)	74.1 (63)
**T (96)**	66.6 (64)	57.2 (55)
**U (108)**	43.5 (47)	35.2 (38)
**Total (735)**	68.4 (503)	61.2 (450)

Legend: DoL, day of life; neonatal centers (n), number (n) of MP infants included during the study period; total proteinintake > 2.5g/kg/day correspond to a minimal protein intake to be reached at the end of the first week according to international recommendations [van Goudoever et al., *Clinical Nutr*, 2018] expressed as the percentage of MP infants reaching the target; total energy intake > 90 kcal/kg/day correspond to a minimal energy intake to be reached at the end of the first week according to international recommendations [Joosten et al Clinical Nutrition 2018] expressed as the percentage of MP infants reaching the target.
